# Moving from human-centred to society-centred design of health technologies

**DOI:** 10.1093/eurpub/ckac129.149

**Published:** 2022-10-25

**Authors:** J Niess

**Affiliations:** 1 Human-Computer-Interaction, University of St. Gallen, St. Gallen, Switzerland; 2 Leibniz ScienceCampus Digital Public Health, Bremen, Germany

## Abstract

The digitised society promises technological solutions to support mental and physical health and well-being. Amongst others, the consumer market, health insurance providers, and companies offer technologies to, among other things, track one's mood, improve diet and fitness habits, foster healthy sleep patterns or track our brain waves to enhance relaxation. However, while the potential benefits of such technologies are apparent and technologies might increase individual and public health, long-term engagement with health technologies is comparably low. This hints at ineffective solutions and insufficient knowledge of user needs. Understanding the specific context of use and user needs is vital to increasing technology adoption, personal benefits, and profitability of health technologies. Taking the user perspective into account when designing technologies is essential to support health and well-being. To foster long-term engagement with health technologies, we argue for considering the broader social context of digital health tools and reflecting on ways how we can empower society to design better and more inclusive health technologies. Hence, when developing digital public health tools, it is essential to go beyond conceptualising people as users and instead shift the focus to humans as part of society and embed such consideration in the design process.

